# Medical Appointment in the University New York

**Published:** 1847-09

**Authors:** 


*Medical Appointment in the University of New-York*—We learn from the Boston Medical and Surgical Journal, that Samuel H. Dickson, M. D., of Charleston, S. C., has been appointed to the chair in the University of the city of New-York, rendered vacant by the decease of the lamented Revere.

Dr. Dickson has for several years occupied the chair of practice, in the Medical College at Charleston, and is favorably known as the author of a work entitled essays on Pathology and Therapeutics.

